# Supplementary material for: Divergent engagements between adeno-associated viruses with their cellular receptor AAVR
Source: Nat Commun. 2019 Aug 21;10:3760. doi: 10.1038/s41467-019-11668-x (PMC6704107; doi:10.1038/s41467-019-11668-x)
Supplement: Supplementary file 1 — Supplementary Information [file 41467_2019_11668_MOESM1_ESM.pdf]

Supplementary Information for:

Divergent engagements between adeno-associated viruses with their cellular receptor AAVR

Ran Zhang, Guangxue Xu, Lin Cao, Zixian Sun, Yong He, Mengtian Cui, Yuna Sun, Shentao Li, Huapeng Li, Lan Qin, Mingxu Hu, Zhengjia Yuan, Zipei Rao, Wei Ding, Zihe Rao and Zhiyong Lou

Contents **Page**

Supplementary Figures 2
Supplementary Figure 1 2
Supplementary Figure 2 3
Supplementary Figure 3 5
Supplementary Figure 4 7
Supplementary Figure 5 8
Supplementary Figure 6 9
Supplementary Figure 7 12
Supplementary Figure 8 13
Supplementary Figure 9 15
Supplementary Figure 10 17
Supplementary Figure 11 19
Supplementary Figure 12 20
Supplementary Figure 13 21
Supplementary Tables 23
Supplementary Table 1. Cryo-EM data statistics 23
Supplementary Table 2. Secondary structure assignment of the AAV5 capsid 25
Supplementary Table 3. Interaction between AAV5 and PKD1 26
Supplementary Table 4. Secondary structure assignment of the AAV1 capsid 27
Supplementary Table 5. Interaction between AAV1 and PKD2 28
Supplementary Table 6. Assignment of variable regions in the AAV5 capsid 29
Supplementary Table 7. Assignment of variable regions in the AAV1 capsid 29
Supplementary Table 8. Primers for AAV capsid mutation cloning 30
Supplementary Table 9. Primers for AAVR mutation cloning 32

Supplementary Figures

Supplementary Figure 1

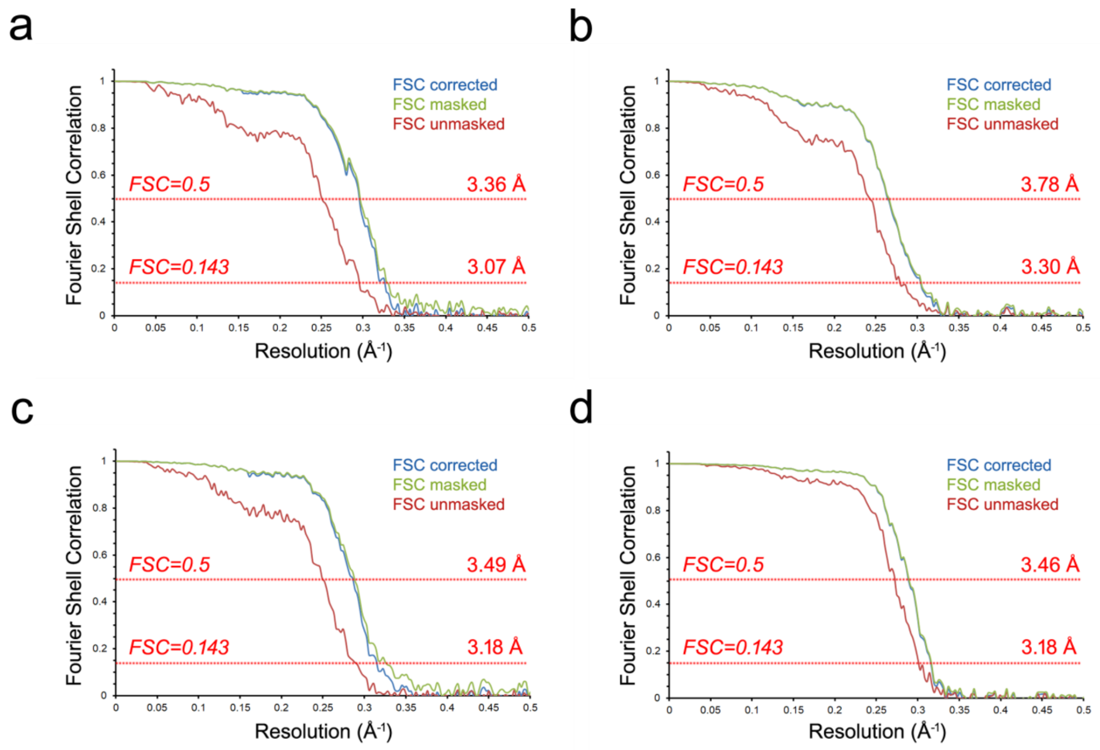

Supplementary Figure 1. Resolution assessment. Fourier shell correlation (FSC) of the final 3D reconstruction following gold standard refinement using RELION and THUNDER. The resolutions corresponding to an FSC of 0.143 are shown for unbound AAV1 **(a)**, the AAV1-AAVR complex **(b)**, unbound AAV5 **(c)** and the AAV5-AAVR complex **(d)**. FSC curves are plotted before (red) and after (green) masking in addition to postcorrection (blue), accounting for the effect of the mask using phase randomization.

Supplementary Figure 2

a

b

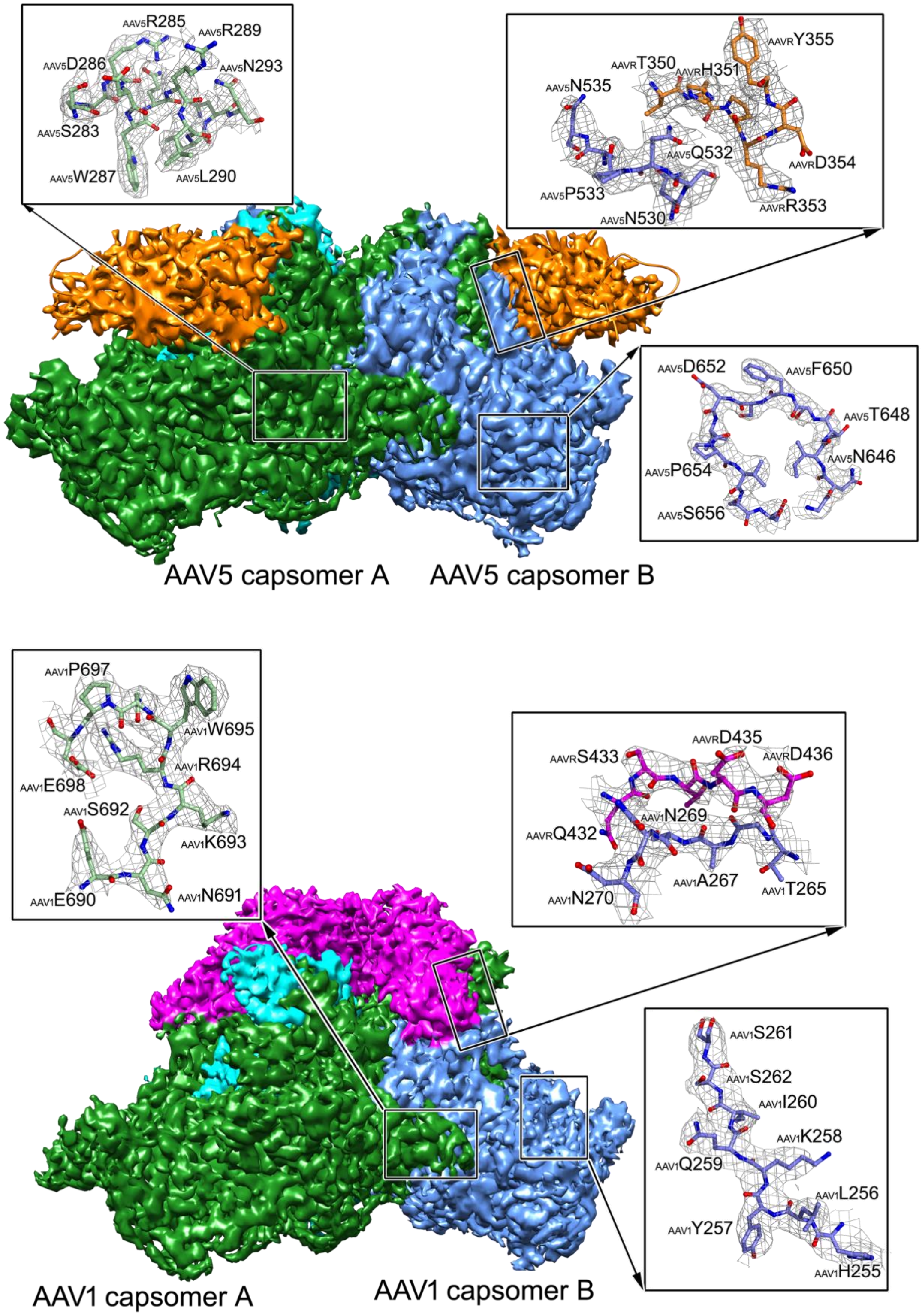

Supplementary Figure 2. Density maps of the AAV5-AAVR and AAV1-AAVR complexes. Shaded surface representation of the density maps for the trimeric AAV5 **(a)** or AAV1 **(b)** capsomers in complex with three molecules of PKD1 (in gold) **(a)** or PKD2 (in magenta) **(b)**. The densities for three AAV1 and AAV5 capsomers are colored green, blue and cyan, respectively, in both panels. In the surrounding boxes, atomic models shown as sticks are superimposed to indicate the representative regions in wire frames. In the stick models, the residue numbers are indicated. The AAVR, AAV1 and AAV5 residues are labeled with a subscript.

Supplementary Figure 3

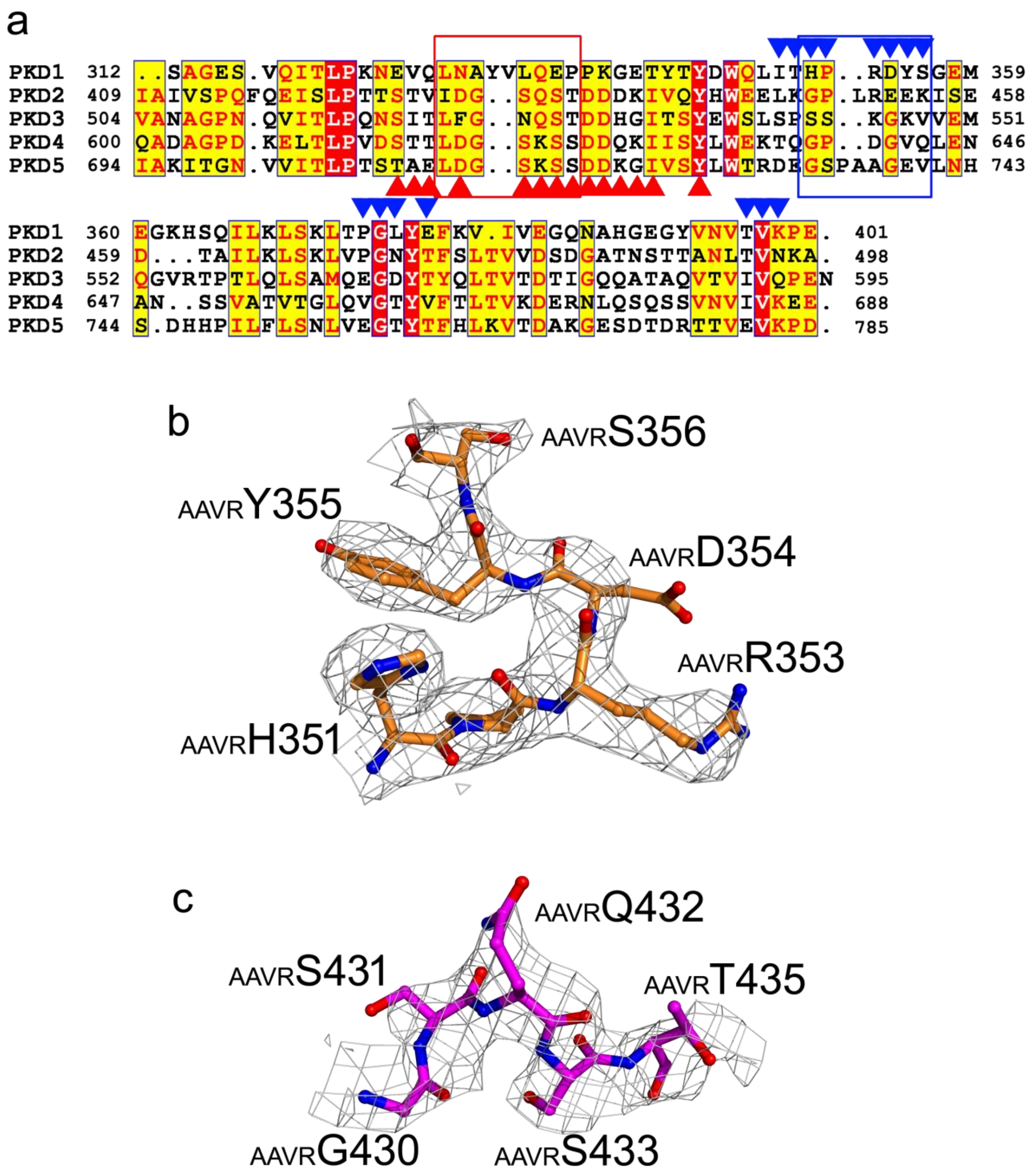

Supplementary Figure 3. (a) Sequence alignment of AAVR PKD1-5. Residues with red or yellow backgrounds are identical or conserved, respectively. Residues in PKD1 and PKD2 at the AAV5-AAVR and AAV1-AAVR interfaces are

indicated with blue and red triangles, respectively. Residues in PKD1 and PKD2 spanning the density in panel **b** and panel **c** are highlighted in blue and red boxes, respectively. **(b)** The residues in a featured fragment from PKD1 ($_{AAVR}$H351-$_{AAVR}$S356) are shown as colored sticks and surrounded by density. The large densities of the side chains of $_{AAVR}$H351 and $_{AAVR}$Y355, and short density of the side chain at position $_{AAVR}$S356 are different from those for their counterparts in other PKDs. **(c)** The residues in a featured fragment from PKD2 ($_{AAVR}$G430-$_{AAVR}$T435) are shown as colored sticks and surrounded by density. The short density of the side chain at position $_{AAVR}$S433 distinguishes it from an asparagine residue in PKD3 and a leucine residue in PKD1.

Supplementary Figure 4

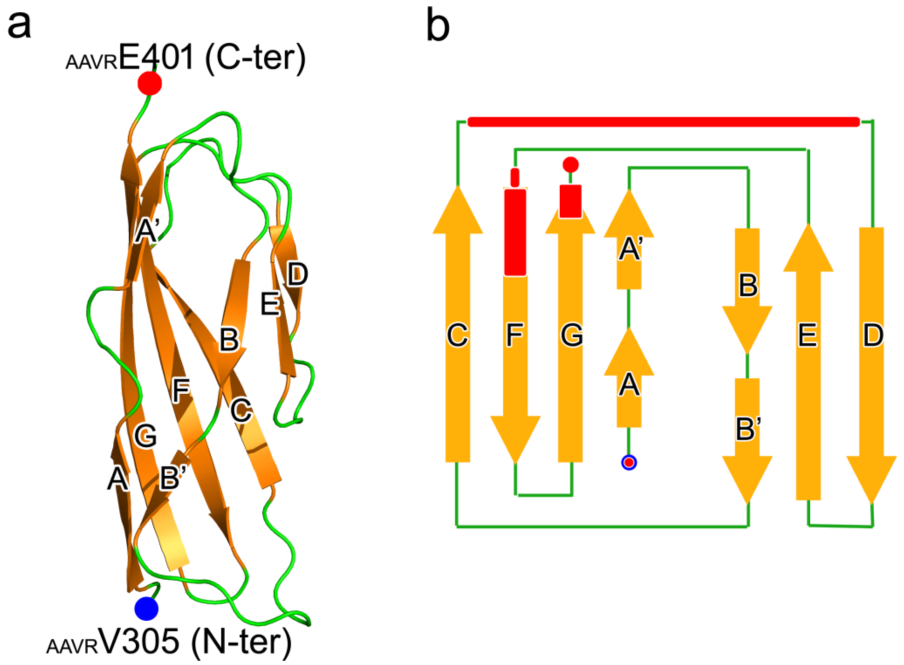

Supplementary Figure 4. Structure of AAVR PKD1 bound to AAV5. (a) PKD1 adopts an Ig-like fold and contains nine β-strands labeled A-G and colored in orange. The loops between each β-strand are shown in green. **(b)** Topological secondary structures of PKD1. The β-strands are alphabetically labeled and shown as orange arrows. The regions that interact with AAV5 are highlighted by red frames. The N-terminal residue, $_{AAVR}$V305, is also involved in the interaction with AAV5 and is highlighted with a red dot.

Supplementary Figure 5

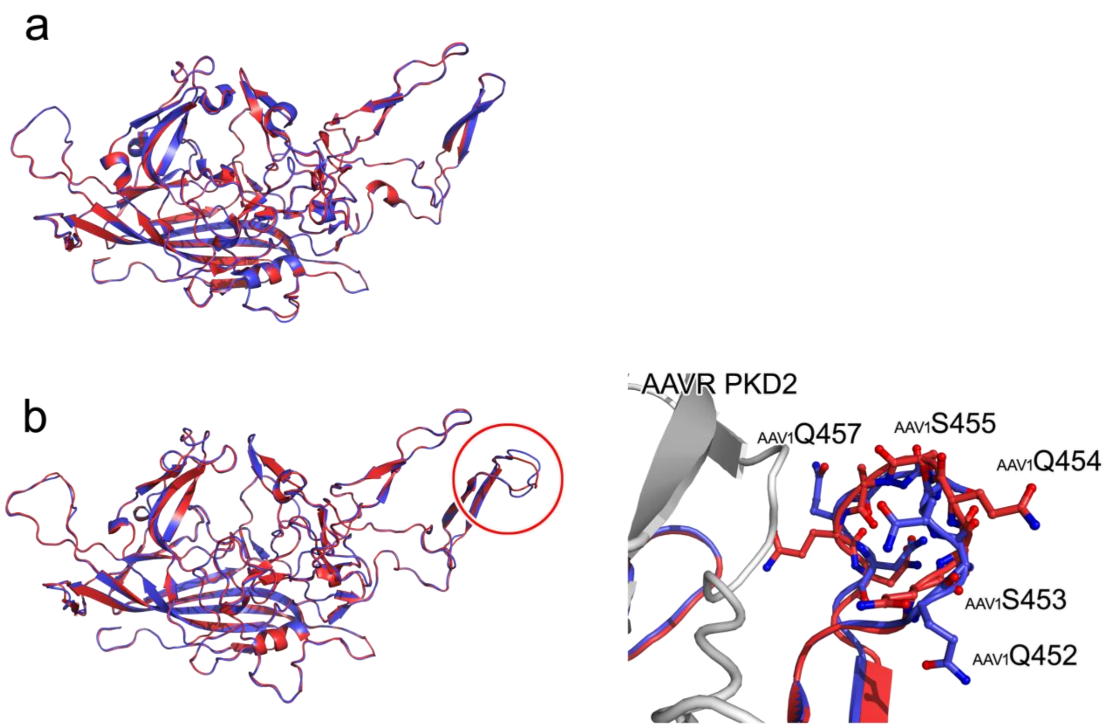

Supplementary Figure 5. Structural alignment of unbound and AAVR-bound AAV5 and AAV1. The structures of the AAV5 capsomer in its unbound form (blue) and in an AAVR-bound state (red) **(a)** and the AAV1 capsomer in its unbound form (blue) and in an AAVR-bound state (red) **(b)** are shown in cartoon representations and aligned individually. The region showing a significant conformational shift in the AAVR-bound AAV1 capsomer is highlighted by a red circle and enlarged in the right panel of **(b)**.

Supplementary Figure 6

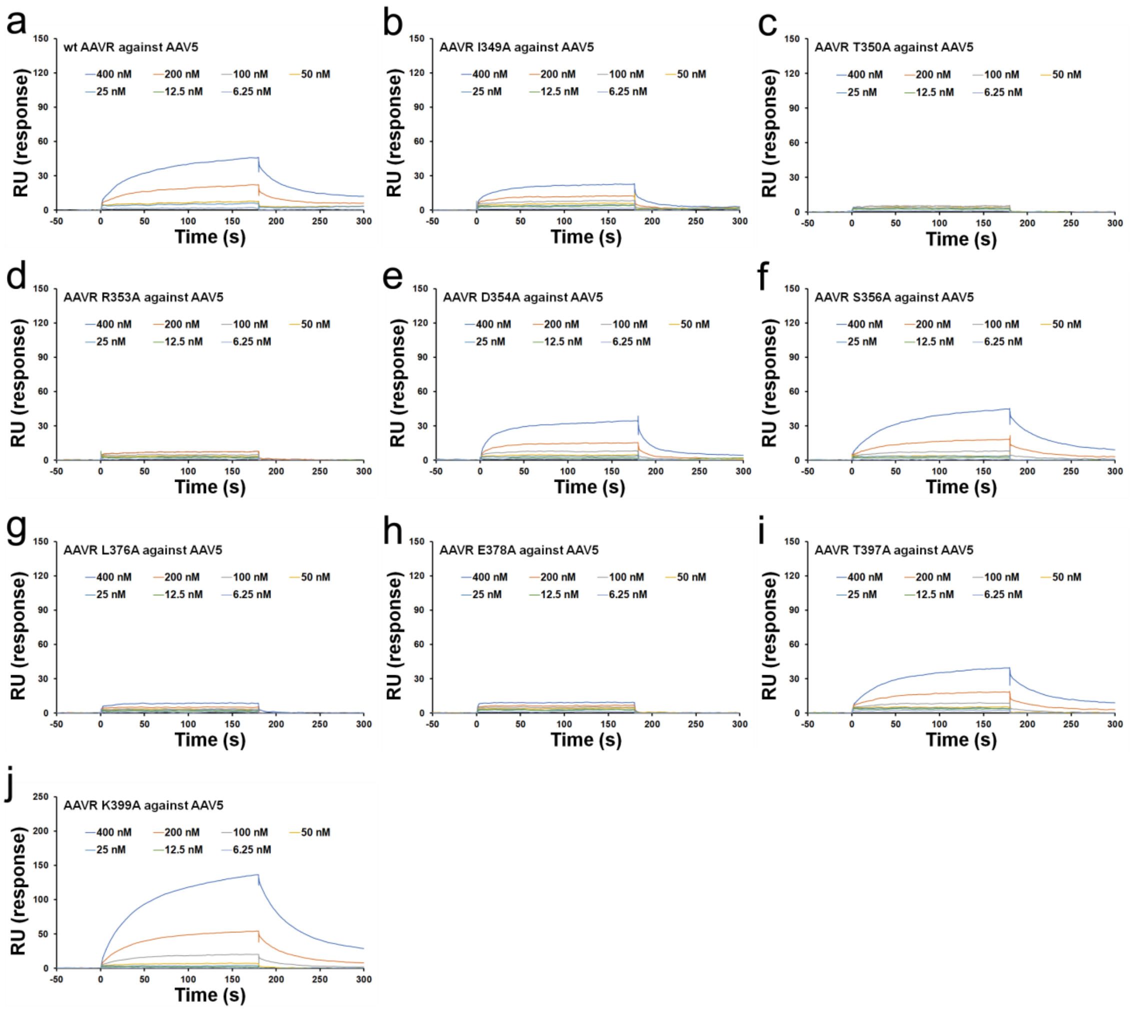

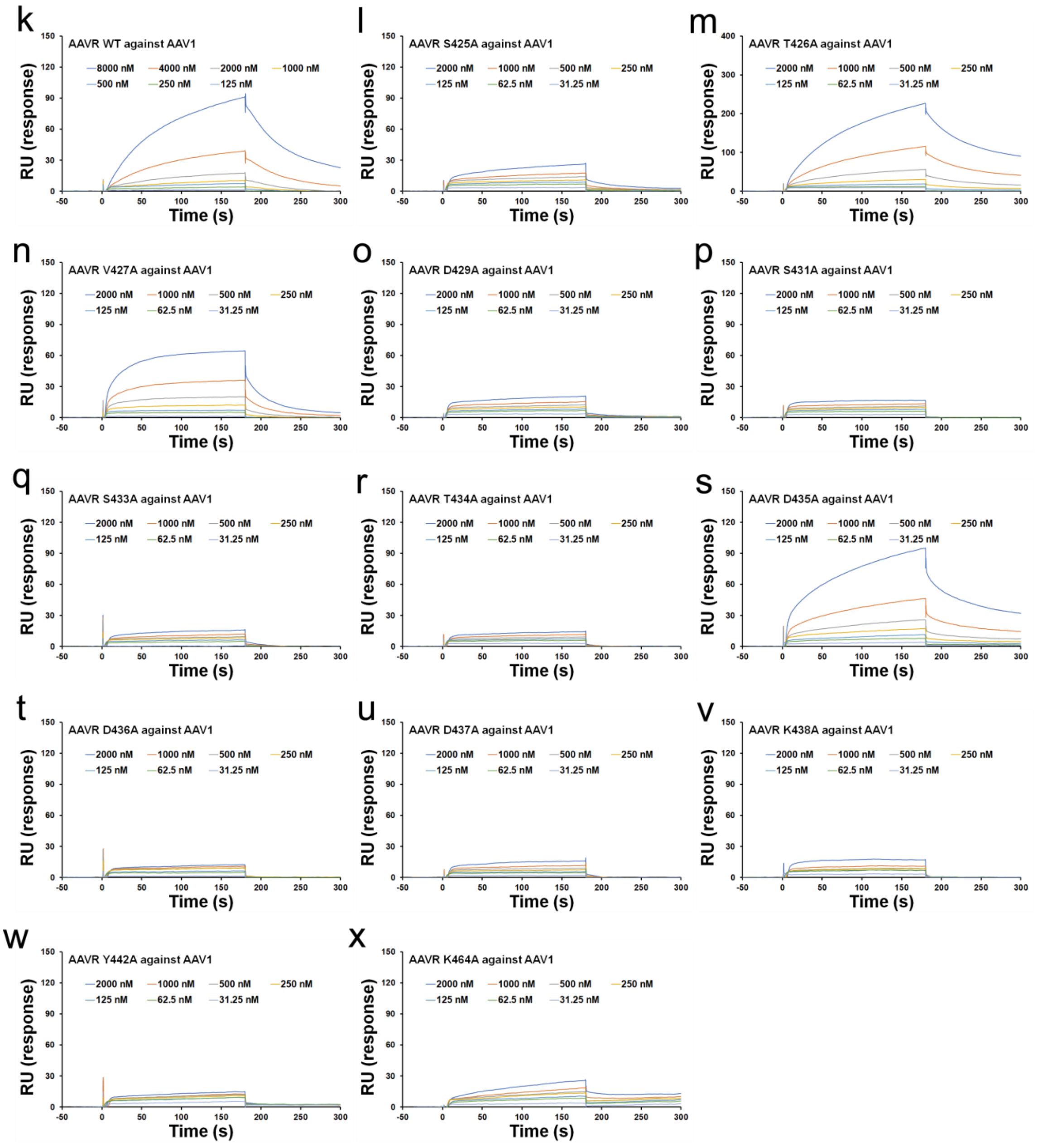

Supplementary Figure 6. Mutagenesis study of AAVR for AAV5 and AAV1 binding assays. (a-j) A total of nine PKD1 mutants were tested for their ability to bind to AAV5 by BIAcore sensorgrams. **(k-x)** A total of thirteen PKD2 mutants were tested for their ability to bind to AAV5 by BIAcore sensorgrams. The concentrations of the analytes are indicated in each panel. The analytes with RU

values under 20 for the highest concentration tested are denoted as nondetectable (N.D.). Each panel is a representative for triplicate experiments.

Supplementary Figure 7

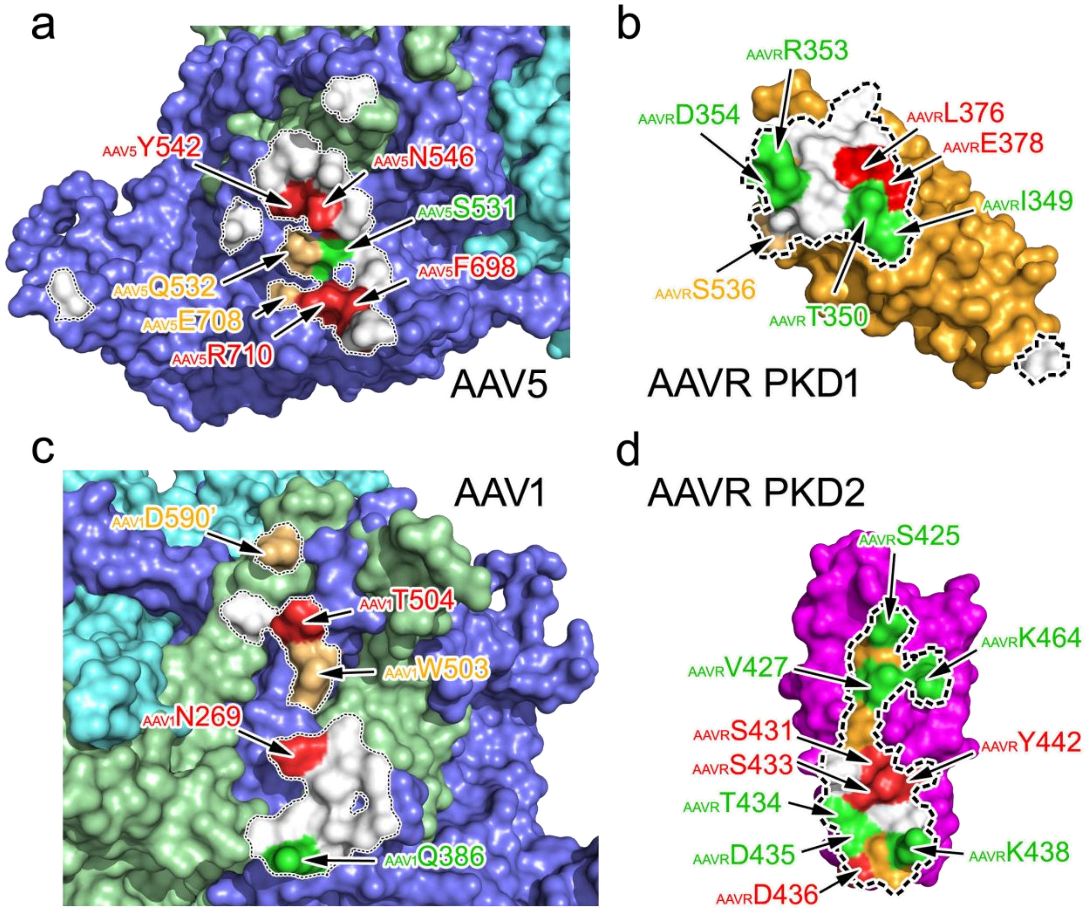

Supplementary Figure 7. Different impacts of the interacting residues on AAV5 and AAV1 transduction. The interacting regions at the virus-receptor interface on the AAV5 capsid **(a)** and AAVR PKD1 **(b)**, the AAV1 capsid **(c)** and AAVR PKD2 **(d)** are framed with dashed lines. Three AAV capsomers of AAV5 **(a)** and AAV1 **(c)** are covered with blue, green and cyan surfaces. AAVR PKD1 **(b)** and PKD2 **(d)** bound to AAVs are colored orange and magenta, respectively. The interacting residues with the greatest impact, a mild impact, or a negligible impact on viral transduction are colored red, light orange and white, respectively. Residues that increased viral transduction are colored green. Residues whose side chains were not involved in the interaction with AAVR are colored white.

Supplementary Figure 8

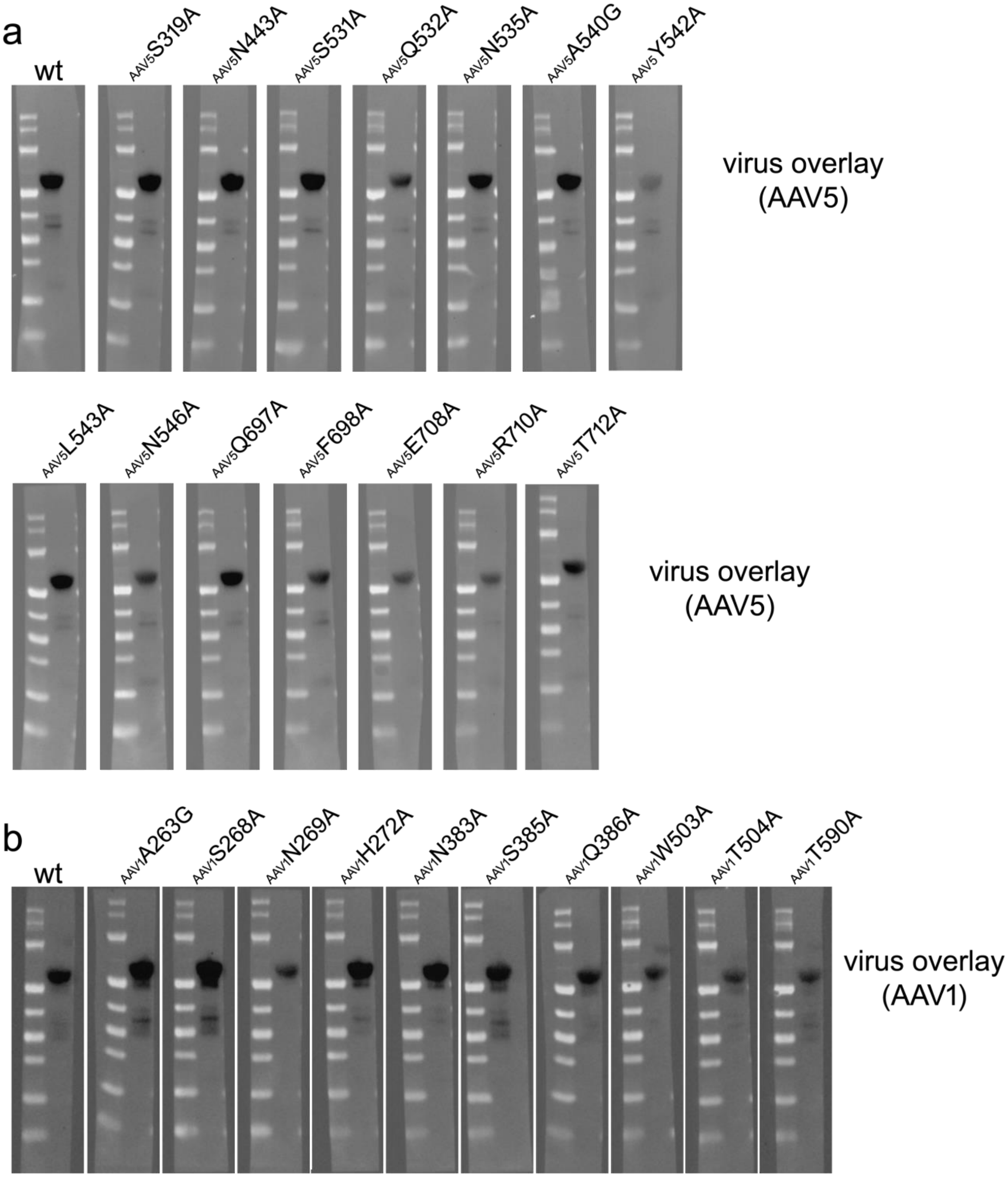

Supplementary Figure 8. Virus overlay assays. Equal amount (6 μg) of wt AAVR was loaded onto Bis-Tris gels, and virus overlay assays with AAV5 **(a)** or AAV1 **(b)** and the related mutations were performed. The molecular weights for

standard protein makers are 180 kDa, 130 kDa, 95 kDa, 55 kDa, 43 kDa, 34 kDa, 26 kDa, 17 kDa and 10 kDa from top to bottom, respectively.

Supplementary Figure 9

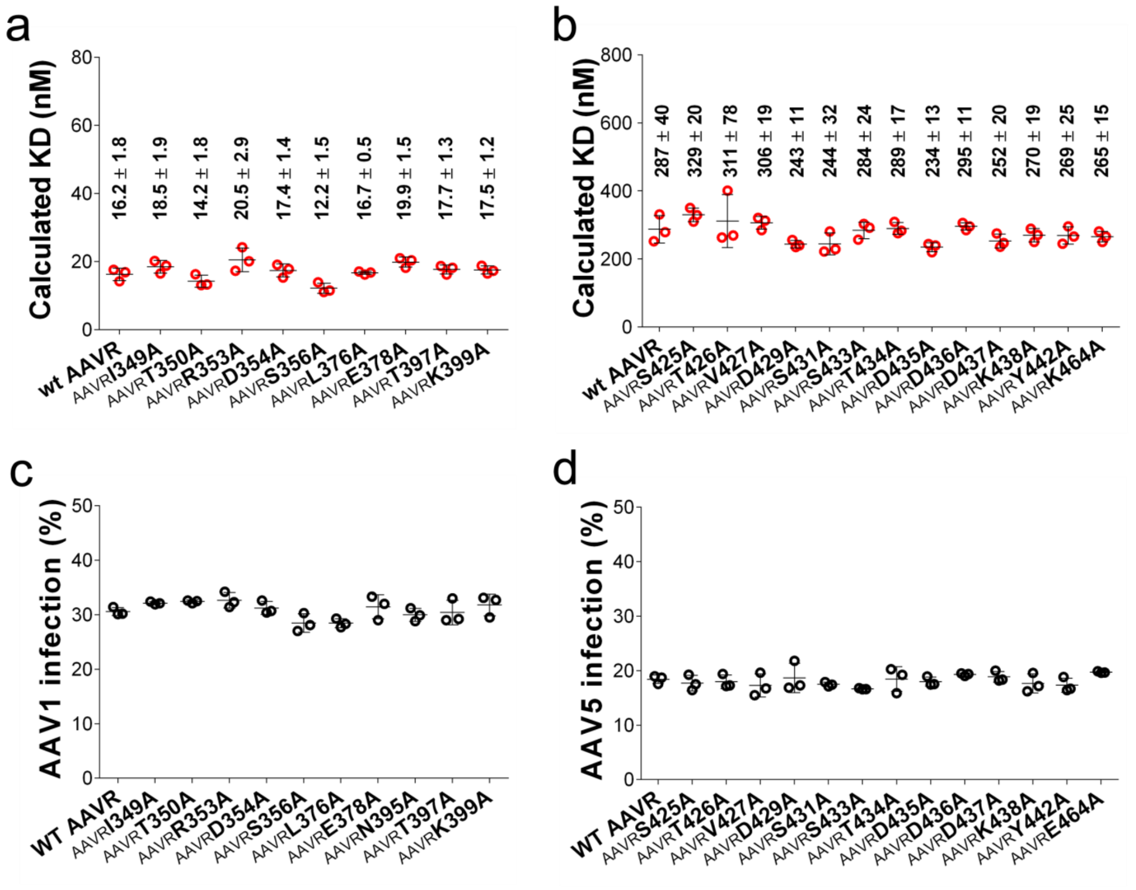

Supplementary Figure 9. (a) A total of nine AAVR PKD1 mutants were tested for their ability to bind AAV1 by BIAcore sensorgrams in triplicate experiments. **(b)** A total of thirteen AAVR PKD2 mutants were tested for their ability to bind AAV5 by BIAcore sensorgrams in triplicate experiments. The calculated KD values for the binding of each mutant to virus are summarized as the mean values of three experiments with standard errors. **(c)** The impact of AAVR PKD1 mutants overexpressed in AAVR-silenced HEK293T cells on AAV1 transduction. Cells were transfected with wt AAVR or different AAVR mutants as indicated followed by infection with AAV1-mCherry at an MOI of 5×10^5 vg per cell. **(d)** The overexpression of AAVR PKD2 mutants in AAVR-silenced HEK293T cells impacted on AAV5 transduction. Cells were transfected with wt AAVR or different

AAVR mutants as indicated followed by infection with AAV5-mCherry at an MOI of 3×10^6 vg per cell. The percentage of mCherry positive cells are plotted as means +/- standard errors (n=3). Source data are provided as a Source Data file.

Supplementary Figure 10

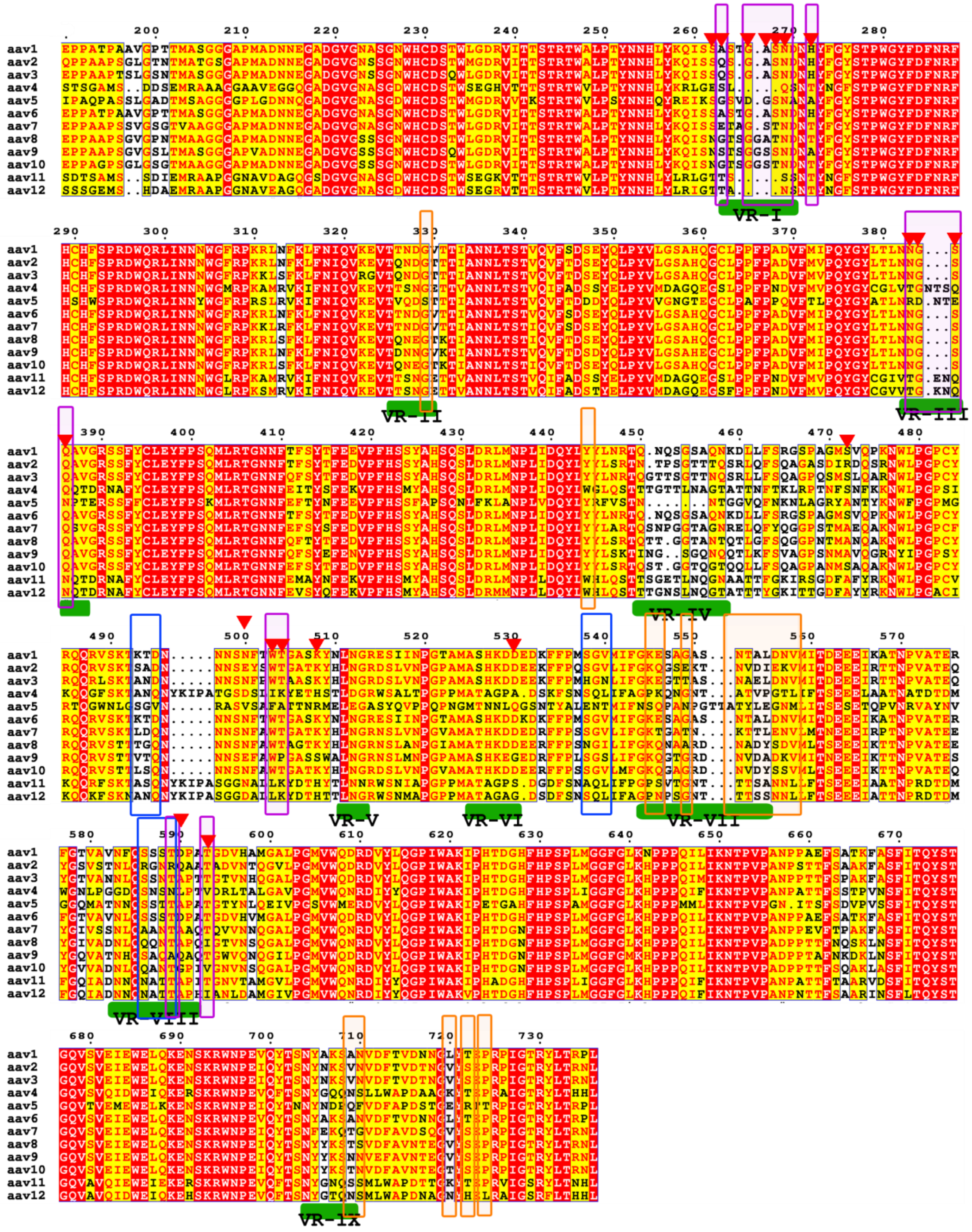

Supplementary Figure 10. Sequence alignment of the VP3 region of the AAV1-AAV12 capsids. The labeled numbers correspond to the amino acid

residues in the AAV1 VP1 coding region. The variable regions between different AAV serotypes are colored green and labeled. Residues that interact with HPSG analog in AAV2, PKD2 in AAV1, and PKD1 in AAV5 are highlighted in blue, magenta and orange frames, respectively. Residues that contact PKD2 from AAV2 are marked with red stars. Identical or conserved residues are shown as letters with red or yellow backgrounds, while the nonconserved residues have a white background.

Supplementary Figure 11

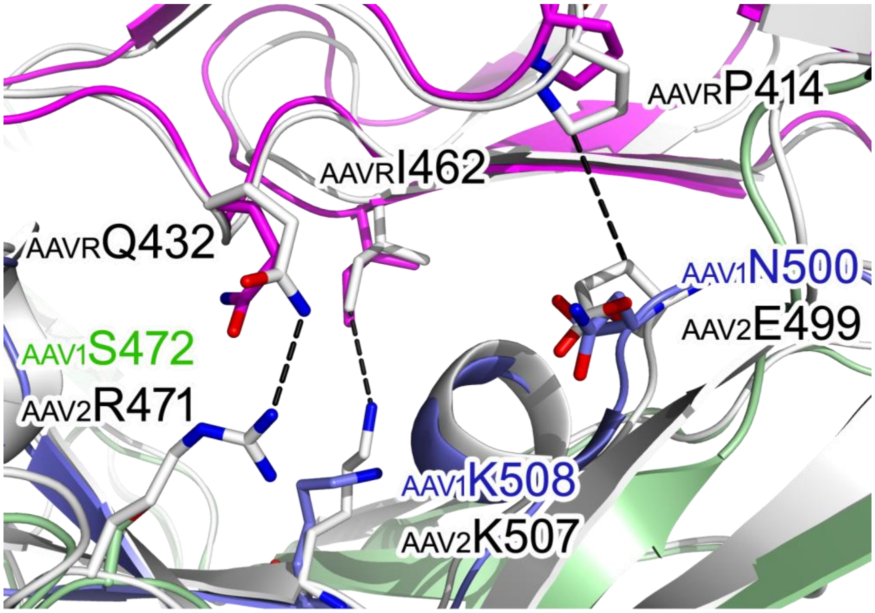

Supplementary Figure 11. Three positions are involved in the PKD2-AAV2 interaction but not the PKD2-AAV1 interaction. The AAV1-AAVR PKD2 and AAV2-AAVR PKD2 complex structures are aligned referenced by the polypeptides of the AAV1 and AAV2 capsomers. Two AAV1 capsomer polypeptides are shown as blue and green cartoons, while two AAV2 capsomer polypeptides are displayed as white cartoons. The PKD2 molecules bound to AAV1 and AAV2 are represented as magenta and while cartoons, respectively. Three positions in the AAV1 and AAV2 capsids with distinct interactions with AAVR PKD2, as well as the interacting residues in AAVR PKD2 are shown as colored sticks with labels. Dashed lines denote bonds with a distance less than 3.5 Å.

Supplementary Figure 12

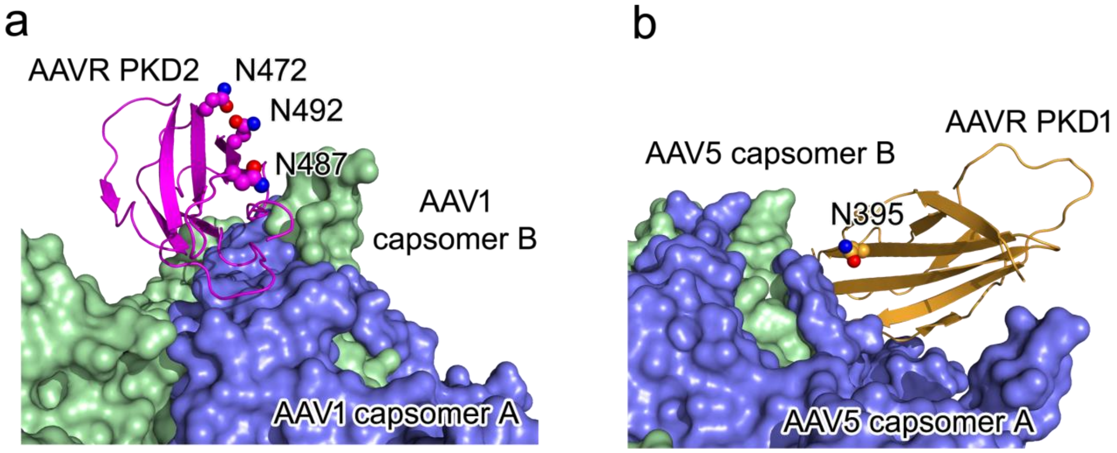

Supplementary Figure 12. Glycosylated sites in AAVR. Among the five reported glycosylated sites in AAVR, three are found in the structure of PKD2 bound to AAV1 **(a),** and one is located in a PKD1 molecule bound to AAV5 **(b)**. The two AAV1 or AAV5 capsomers are shown as blue and green surfaces, while the bound PKD1 and PKD2 are shown as orange and magenta cartoons, respectively. The reported glycosylated residues are highlighted as colored spheres.

Supplementary Figure 13

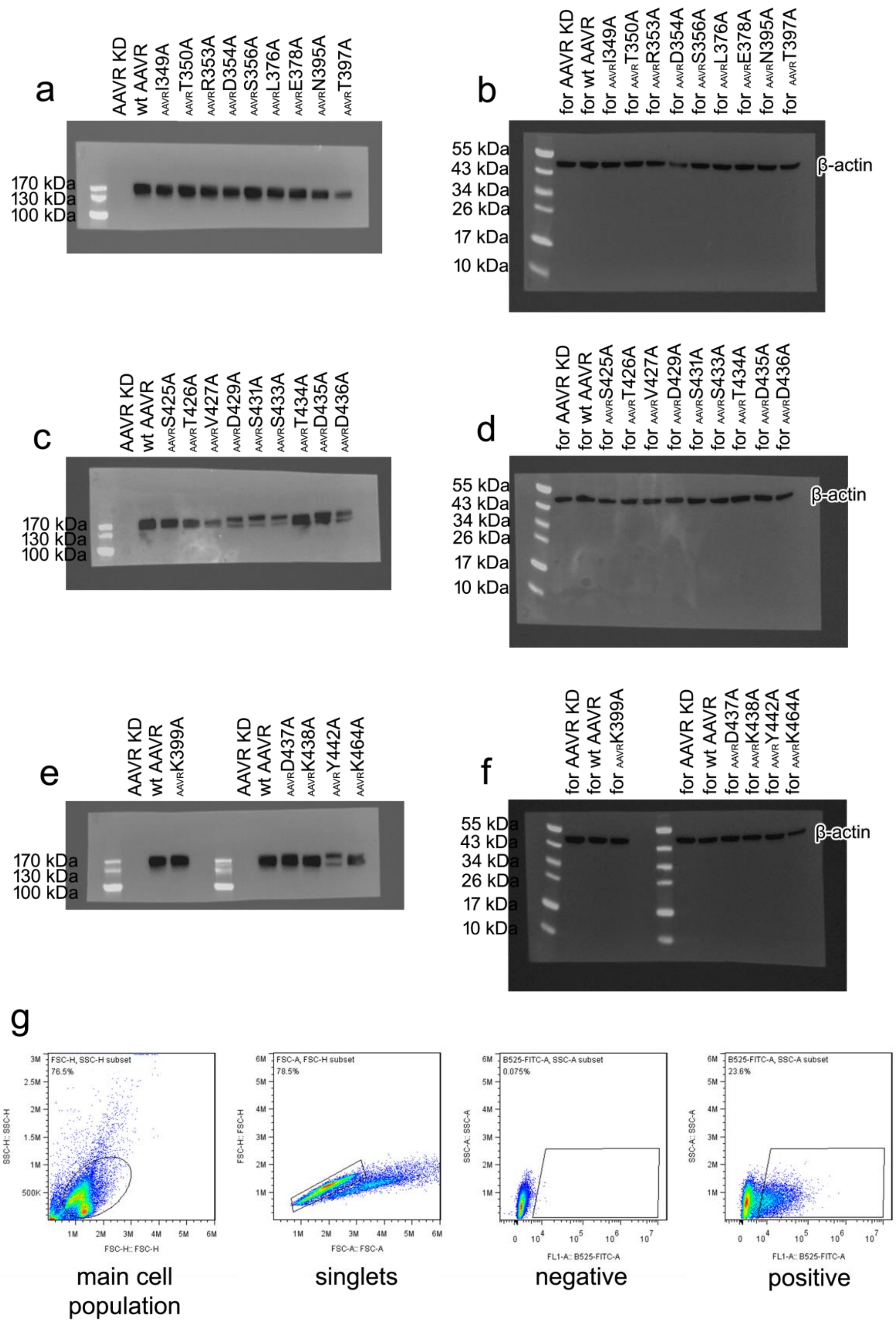

Supplementary Figure 13. Full-length gels and immunoblots. (a, c, e) Whole gels showing the expression of wt AAVR or AAVR mutants in HEK293T cells with shRNA. After blocking, membranes were cut to 2 halves along the 72 kDa protein marker. AAVR was immunoblotted with anti-AAVR antibodies in the upper half. The corresponding samples were loaded onto another gel, and β-actin was immunoblotted as a control in the lower half **(b, d, f)**. The molecular weights of AAVR and β-actin are 150 kDa and 43 kDa, respectively. The molecular weights of standard protein markers are labeled in each panel. **(g)** Illustration of gating strategy of flow cytometry.

Supplementary Tables

Supplementary Table 1. Cryo-EM data statistics

	AAV1 alone EMD-9795, PDB 6JCR	AAV1-AAVR EMD-9794, PDB 6JCQ	AAV5 alone EMD-9797, PDB 6JCT	AAV5-AAVR EMD-9796, PDB 6JCS
**Data collection and processing**				
Magnification	110,000	110,000	110,000	110,000
Voltage (kV)	200	200	200	200
Electron exposure (e–/Å^2)	25.04	25.04	25.04	25.04
Defocus range (μm)	-2.5 to -1.2	-2.5 to -1.2	-2.5 to -1.2	-2.5 to -1.2
Pixel size (Å)	0.93	0.93	0.93	0.93
Symmetry imposed	I1	I1	I1	I1
Initial particle images (no.)	3,499	3,831	3,545	14,221
Final particle images (no.)	2,642	2,926	2,900	12,590
Map resolution (Å)	3.07	3.30	3.18	3.18
FSC threshold	0.143	0.143	0.143	0.143
Map resolution range (Å)	2.6-3.07	2.7-3.3	2.7-3.18	2.7-3.18
**Refinement**				
Initial model used (PDB code)	5EG3	5EG3, 6IHB	3NTT	3NTT, 2E7M
Model resolution (Å)	3.07	3.30	3.18	3.18
FSC threshold	0.143	0.143	0.143	0.143
Model resolution range (Å)	∞ to 3.07	∞ to 3.30	∞ to 3.18	∞ to 3.18
Map sharpening *B* factor (Å^2)	-132.4	-159.3	-148.00	-152.8
Correlation coefficient between map and model	0.824	0.815	0.825	0.811
Model composition				
Non-hydrogen atoms	4,112	4,830	4,110	4,881
Protein residues	517	611	515	611
Ligands	0	0	0	0
B factors (Å^2)				
Protein	32.9	36.7	35.1	33.8
Ligand	--	--	--	--
R.m.s. deviations				
Bond lengths (Å)	0.009	0.009	0.008	0.010
Bond angles (°)	1.030	0.971	0.970	1.008
Validation				
MolProbity score	1.68	1.97	1.69	1.79
Clashscore	4.88	6.27	4.39	5.90
Poor rotamers (%)	0.22	0.19	0.44	0.56
Ramachandran plot				
Favored (%)	93.60	89.46	92.61	92.45

Allowed (%)	6.40	10.21	7.20	7.22
Disallowed (%)	0	0.33	0.19	0.33

Supplementary Table 2. Secondary structure assignment of the AAV5 capsid

No.	Secondary Structure	Range of Residues		No.	Secondary Structure	Range of Residues
**1**	βA	221-225		**22**	βGH5	446-451
**2**	βB	228-239		**23**	βGH6	469-470
**3**	αBC1	241-245		**24**	βGH7	474-475
**4**	βBC2	248-252		**25**	αGH8	488-492
**5**	αBC3	258-262		**26**	βGH9	494-497
**6**	βC	263-272		**27**	βGH10	500-503
**7**	αCD1	277-281		**28**	βGH11	511-512
**8**	αA	283-293		**29**	βGH12	521-522
**9**	βD	294-315		**30**	βGH13	528-530
**10**	βDE1	322-324		**31**	βGH14	547-549
**11**	βE	331-336		**32**	αGH15	552-556
**12**	αEF1	344-348		**33**	βGH16	560-561
**13**	βEF2	361-362		**34**	βGH17	567-570
**14**	βEF3	365-370		**35**	βGH18	582-585
**15**	αEF4	387-391		**36**	βGH19	608-610
**16**	βF	394-396		**37**	βGH20	627-629
**17**	βG	402-407		**38**	βH	635-640
**18**	βGH1	414-415		**39**	βI	661-677
**19**	βGH2	418-419		**40**	βI1	702-703
**20**	αGH3	423-427		**41**	βI2	709-710
**21**	βGH4	436-442		**42**	βI3	721-722

The secondary structures of AAV5 capsid are assigned as a previously reported crystallographic AAV5 structure (PDB code: 3NTT).

Supplementary Table 3. Interaction between AAV5 and PKD1

AAVR Residues	Contacts[1]	AAV5 Residues
V305	1	S319
I349	1	N535
T350	4	Q532
H351	4, 2, 4, 8	Q532, N546, E708, R710
P352	5, 4	G545, N546
R353	2, 6, 2, 1, 4	S531, G545, M547, F698, T712
D354	1	T712
Y355	2, 4	F698, R710
S356	3	Q697
P374	1	G545
G375	3	L543
L376	1, 2, 1, 6	A540, T541, Y542, N546
E378	1	A540
T397	1	L543
V398	1	L543
K399	3	N443

[1]Numbers represent the number of atom-to-atom contacts between the AAVR residues and the AAV5 residues, analyzed by the Contact program in the CCP4 suite (with a distance cutoff of 4 Å).

Supplementary Table 4. Secondary structure assignment of the AAV1 capsid

No.	Secondary Structure	Range of Residues		No.	Secondary Structure	Range of Residues
**1**	βA	232-235		**17**	βGH2	443-449
**2**	βB	238-249		**18**	βGH3	460-465
**3**	αBC1	253-255		**19**	βGH4	487-490
**4**	βBC2	258-262		**20**	αGH5	501-504
**5**	βC	273-282		**21**	βGH6	508-511
**6**	αCD1	288-290		**22**	βGH7	514-516
**7**	αA	294-302		**23**	βGH8	533-535
**8**	βD	304-325		**24**	βGH9	542-544
**9**	βDE1	330-334		**25**	αGH10	563-567
**10**	βE	342-346		**26**	βGH11	578-581
**11**	βEF1	371-373		**27**	βGH12	593-596
**12**	βEF2	375-381		**28**	βGH13	619-621
**13**	αEF3	396-398		**29**	βGH14	638-640
**14**	βF	401-405		**30**	βH	647-651
**15**	βG	409-416		**31**	βI	673-689
**16**	βGH1	426-428		**32**	βI1	732-734

The secondary structures of AAV1 capsid are referenced with the structure of AAV2 (PDB code: 1LP3).

Supplementary Table 5. Interaction between AAV1 and PKD2

AAVR Residues	Contacts[1]	AAV1 Residues
S425	9	D590
T426	1	D590
V427	2	T504
D429	1, 5	T504, W503
S431	1	W503
Q432	2, 1	S268, W503
S433	1, 1	S268, N269
T434	1, 6, 5	G266, A267, S268
D435	3, 5, 3	G266, A267, H272
D436	3, 2, 3	A263, H272, S385
D437	6, 1, 5	H272, S385, Q386
K438	1, 2, 1	N269, N383, G384
I439	1	N269
Y442	2	N269
K464	1	T593

[1]Numbers represent the number of atom-to-atom contacts between the AAVR residues and the AAV1 residues, analyzed by the Contact program in the CCP4 suite (with a distance cutoff of 4 Å).

Supplementary Table 6. Assignment of variable regions in the AAV5 capsid

No.	Name of variable region	Corresponding secondary structure	Range of residues	in AAVR interaction?
1	VR-I	loop βBC2-αBC3	253-258	No
2	VR-II	loop βD-βDE1	315-321	Yes
3	VR-III	loop βEF2-αEF4	370-381	No
4	VR-IV	loop βGH4-βGH5	442-446	No
5	VR-V	loop βGH9-βGH10	497-500	No
6	VR-VI	loop βGH11-βGH12	514-520	No
7	VR-VII	loop βGH13-βGH14	536-546	Yes
8	VR-VIII	loop βGH17-βGH18	572-581	No
9	VR-IX	loop βI-βI1	693-698	Yes

Supplementary Table 7. Assignment of variable regions in the AAV1 capsid

No.	Name of variable region	Corresponding secondary structure	Range of residues	in AAVR interaction?
1	VR-I	loop βBC2-βC	263-265	Yes
2	VR-II	loop βD-βDE1	326-330	No
3	VR-III	loop βEF2-αEF3	381-388	Yes
4	VR-IV	loop βGH2-βGH3	449-457	No
5	VR-V	loop βGH6-βGH7	511-513	No
6	VR-VI	loop βGH7-βGH8	525-530	No
7	VR-VII	loop βGH9-αGH10	544-554	No
8	VR-VIII	loop βGH11-βGH12	582-591	Yes
9	VR-IX	loop βI-βI1	704-709	No

Supplementary Table 8. Primers for AAV capsid mutations cloning

Primer Name	Sequence (5'-3')
**cap5-F**	gcgcagccatcgacgtcagac
**cap5-319A-R**	gatggtggtggtggcgtcctgcaccg
**cap5-443A-R**	ctggactccgccagtggcatttgtgctcac
**cap5-531A-R**	gggttcgccggctgggcgttgaagatcatag
**cap5-532A-R**	gcccgggttcgccggggcgctgttgaagatcat
**cap5-535A-R**	gtggtgcccggggccgccggctggctgttg
**cap5-540A-R**	gccctcgaggtacgtgccggtggtgcccgggt
**cap5-542A-R**	atgttgccctcgagggccgtggcggtggtg
**cap5-543A-R**	agcatgttgccctcggcgtacgtggcggtg
**cap5-546A-R**	ctggtgatgagcatggcgccctcgaggtac
**cap5-697A-R**	ggcaaagtccacaaaggcggggtcgttgtagtt
**cap5-698A-R**	cggggcaaagtccacggcctgggggtcgttgta
**cap5-708A-R**	ctggtggttctgtaggccccggtgctgtccg
**cap5-710A-R**	gataggtctggtggtggcgtattccccggtgct
**cap5-712A-R**	cgataggtctggcggttctgtattcccc
**cap5-R**	gaattccagcacactggcggccgttactagtggatcctagagcatggaaactagataag
**cap5-319A-F**	cggtgcaggacgccaccaccaccatc
**cap5-443A-F**	gtgagcacaaatgccactggcggagtccag
**cap5-531A-F**	ctatgatcttcaacgcccagccggcgaaccc
**cap5-532A-F**	atgatcttcaacagcgccccggcgaacccgggc
**cap5-535A-F**	cagccagccggcggccccgggcaccaccgc
**cap5-540A-F**	acccgggcaccaccggcacgtacctcgagggc
**cap5-542A-F**	caccaccgccacggccctcgagggcaacat
**cap5-543A-F**	caccgccacgtacgccgagggcaacatgct
**cap5-546A-F**	gtacctcgagggcgccatgctcatcaccag
**cap5-697A-F**	aactacaacgaccccgcctttgtggactttgcc
**cap5-698A-F**	tacaacgacccccaggccgtggactttgccccg
**cap5-708A-F**	acagcaccggggcctacagaaccaccagac
**cap5-710A-F**	agcaccggggaatacgccaccaccagacctatc
**cap5-712A-F**	gaatacagaaccgccagacctatcggaa
**cap1-F**	gcgcagccatcgacgtcagac
**cap1-263G-R**	ctggcccccgttgagccactggagatttg
**cap1-268A-R**	gtggttgtcgttgGCggcccccgttg
**cap1-269A-R**	gtagtggttgtcgGCgctggcccccg
**cap1-272A-R**	gtagccgaagtagGCgttgtcgttgc
**cap1-383A-R**	cacggcttggctgccGGCgttgagcgtcaggtag
**cap1-385A-R**	cccacggcttggGCgccattgttga
**cap1-386A-R**	tgaacgtcccacggcGGCgctgccattgttgag
**cap1-503A-R**	gttatattttgaagcaccagtGGCggtaaaattgctgttg
**cap1-504A-R**	gttatattttgaagcaccGGCccaggtaaaattgctg
**cap1-590A-R**	ccggtcgcagggGctgtgctgctgc

**cap1-R**	cttgagtccaaagccgcccat
**cap1-263G-F**	caaatctccagtggctcaacgggggccag
**cap1-268A-F**	caacgggggccGCcaacgacaaccac
**cap1-269A-F**	cgggggccagcGCcgacaaccactac
**cap1-272A-F**	gcaacgacaacGCctacttcggctac
**cap1-383A-F**	ctacctgacgctcaacGCCggcagccaagccgtg
**cap1-385A-F**	tcaacaatggcGCccaagccgtggg
**cap1-386A-F**	ctcaacaatggcagcGCCgccgtgggacgttca
**cap1-503A-F**	caacagcaattttaccGCCactggtgcttcaaaatatAAC
**cap1-504A-F**	cagcaattttacctggGCCggtgcttcaaaatataac
**cap1-590A-F**	gcagcagcacagCccctgcgaccgg

Supplementary Table 9. Primers for AAVR mutations cloning

Primer Name	Sequence (5'-3')
**AAVR-349A-F**	ACACCTACGACTGGCAGCTGGCTACTCATCCTA
**AAVR-349A-R**	GCCAGCTGCCAGTCGTAGGTGTAGGTTTCTCC
**AAVR-350A-F**	ACCTACGACTGGCAGCTGATTGCTCATCCTAGA
**AAVR-350A-R**	CAATCAGCTGCCAGTCGTAGGTGTAGGTTTCT
**AAVR-353A-F**	GGCAGCTGATTACTCATCCTGCAGACTACAGTG
**AAVR-353A-R**	GCAGGATGAGTAATCAGCTGCCAGTCGTAGGTG
**AAVR-354A-F**	GCTGATTACTCATCCTAGAGCCTACAGTGGA
**AAVR-354A-R**	GCTCTAGGATGAGTAATCAGCTGCCAGTCGTA
**AAVR-356A-F**	TACTCATCCTAGAGACTACGCTGGAGAAATGG
**AAVR-356A-R**	GCGTAGTCTCTAGGATGAGTAATCAGCTGCC
**AAVR-376A-F**	ATCGAAGCTCACTCCAGGCGC GTATGAATTCA
**AAVR-376A-R**	GCGCCTGGAGTGAGCTTCGATAGTTTGAGGA
**AAVR-378A-F**	AGCTCACTCCAGGCCTGTATGCATTCAAAGTGA
**AAVR-378A-R**	GCATACAGGCCTGGAGTGAGCTTCGATAGTT
**AAVR-395A-F**	CCCATGGGGAAGGCTATGTGGCCGTGACAGTCA
**AAVR-395A-R**	GCCACATAGCCTTCCCCATGGGCATTTTGACCC
**AAVR-397A-F**	GGGAAGGCTATGTGAACGTGGCAGTCAAGCCAG
**AAVR-397A-R**	CCACGTTCACATAGCCTTCCCCATGGGCATTT
**AAVR-399A-F**	GCTATGTGAACGTGACAGTCGCGCCAGAGCCCC
**AAVR-399A-R**	GCGACTGTCACGTTCACATAGCCTTCCCCATG
**AAVR-425A-F**	GATCTCTTTGCCAACCACTGCTACAGTCAT
**AAVR-425A-R**	CAGTGGTTGGCAAAGAGATCTCCTGGAACT
**AAVR-426A-F**	CTCTTTGCCAACCACTTCTGCAGTCATTGA
**AAVR-426A-R**	GAGAAGTGGTTGGCAAAGAGATCTCCTGGAAC
**AAVR-427A-F**	TTGCCAACCACTTCTACAGCCATTGATGGCAG
**AAVR-427A-R**	GCTGTAGAAGTGGTTGGCAAAGAGATCTCCT
**AAVR-429A-F**	AACCACTTCTACAGTCATTGCTGGCAGTCAAAG
**AAVR-429A-R**	GCAATGACTGTAGAAGTGGTTGGCAAAGAGAT
**AAVR-431A-F**	ACTTCTACAGTCATTGATGGCGCTCAAAGCACTGAT
**AAVR-431A-R**	GCGCCATCAATGACTGTAGAAGTGGTTGGCAA
**AAVR-433A-F**	CAGTCATTGATGGCAGTCAAGCCACTGATGATG
**AAVR-433A-R**	GCTTGACTGCCATCAATGACTGTAGAAGTGGT
**AAVR-434A-F**	TCATTGATGGCAGTCAAAGCGCTGATGATGATA
**AAVR-434A-R**	CGCTTTGACTGCCATCAATGACTGTAGAAGTG
**AAVR-435A-F**	TTGATGGCAGTCAAAGCACTGCTGATGATAAAATC
**AAVR-435A-R**	GCAGTGCTTTGACTGCCATCAATGACTGTAGA
**AAVR-436A-F**	ACAGTCATTGATGGCAGTCAAAGCACTGATGCTGATAAAATC
**AAVR-436A-R**	GCATCAGTGCTTTGACTGCCATCAATGACTG
**AAVR-437A-F**	GGCAGTCAAAGCACTGATGATGCTAAAATCGTTCA
**AAVR-437A-R**	GCATCATCAGTGCTTTGACTGCCATCAATGAC
**AAVR-438A-F**	AGTCAAAGCACTGATGATGATGCAATCGTTCAGT

**AAVR-438A-R**	GCATCATCATCAGTGCTTTGACTGCCATCAAT
**AAVR-442A-F**	GATGATGATAAAATCGTTCAGGCCCATTGGGAAG
**AAVR-442A-R**	GCCTGAACGATTTTATCATCATCAGTGCTTTG
**AAVR-464A-F**	CTGAAGATACAGCCATATTAGCACTAAGTAAACTCG
**AAVR-464A-R**	GCTAATATGGCTGTATCTTCAGAAATCTTCTC
